# Comparing placement and polarity configurations of a two-magnet fingertip vibrotactile device

**DOI:** 10.1038/s41598-026-41307-7

**Published:** 2026-03-08

**Authors:** Ifat Gertler, Giulia Ballardini, Demet Tangolar, Gokhan Serhat, Katherine J. Kuchenbecker

**Affiliations:** 1 Haptic Intelligence Department, Max Planck Institute for Intelligent Systems, Heisenbergstr. 3, 70569 Stuttgart, Germany; 2 Faculty of Engineering Design, Production Engineering and Automotive Engineering, University of Stuttgart, Pfaffenwaldring 9, 70569 Stuttgart, Germany; 3 Department of Mechanical Engineering, KU Leuven, Spoorwegstr. 12, 8200 Bruges, Belgium

**Keywords:** Vibrotactile feedback, Wearable haptics, Audio-driven actuation, Phase-shifted vibrations, Finite element analysis, Engineering, Neuroscience

## Abstract

Vibrotactile feedback enriches the use of wearable technologies for entertainment, navigation, and healthcare. The actuators of these portable systems, particularly fingertip devices, need to be compact, comfortable, and easy to integrate. Multiple vibrating elements could enhance perceptual realism, but how should they be arranged and oriented on the fingerpad? Here, we evaluate a simple approach that uses an audio input signal to drive an air coil that vibrates two magnets embedded in a soft fingertip sheath; the magnets are arranged in the radial-ulnar or proximal-distal direction with either the same or opposite polarity. We explore the effects of these new device configurations on both dynamic response and haptic perception. Experimental results indicate that the vibrations were perceived well across frequencies, with stronger sensations between 180 and 360 Hz, which aligns with the high vibration magnitudes our computational simulation predicts in this frequency range. Interestingly, perceptual responses showed that participants mainly classified vibrations based on the excitation frequency rather than the polarity of the magnets. Participants also rated vibrotactile feedback derived from recorded sounds and replayed for different interactions. Their evaluations offer promising evidence that this actuation approach could be used in extended-reality applications to improve transient user interactions with virtual objects.

## Introduction

Soft fingertip vibrotactile devices have gained increasing attention as lightweight, comfortable, and low-cost solutions for delivering haptic feedback^1–3^. Such systems enhance user experience in extended reality^4^ and other settings by providing tactile cues at the expected contact location, enabling rich texture rendering^5^. Furthermore, soft materials enable a stable mechanical coupling between the skin and the actuator^6^, ensuring consistent perception of the delivered signals^7^. However, devices composed solely of soft materials often require complex control and have limited versatility in terms of the frequency or displacement they can deliver^8^. Integrating rigid components into soft robotic devices without compromising their compliant structure can enhance the functionality and increase robustness^9,10^. However, this integration process presents significant challenges in the design and fabrication stages.

Oscillating magnetic fields offer a straightforward approach for generating highly controllable vibrations, making them attractive for soft haptic devices. These fields can be produced by one or more coils to induce oscillations of permanent magnets or ferromagnetic elements, such as particles embedded within a soft matrix^11,12^. Devices can include only one^6,13,14^ or an array of individually actuated magnets^15,16^, providing a versatile strategy for delivering tunable haptic feedback. For such a device to render realistic haptic feedback of object interactions, the coil’s driving signal is often derived from a physical recording of the interaction, such as measurements of the high-frequency acceleration of the interacting body part or tool^17^. While this method provides clean and realistic vibrations, it requires specialized equipment and technical expertise. A more accessible alternative is to use the sound produced by the interaction as the driving signal^18^. Audio recordings are typically single- or two-channel signals whose frequency content falls within the audible range (about 20–20,000 Hz^19^). Consequently, while audio encompasses most of the human vibrotactile sensing range (up to 1,000 Hz^20^), it omits low frequencies and contains acoustic components that do not correspond to tactile information (e.g., harmonics and ambient noise). The inclusion of environmental noise can reduce the vibrotactile signal-to-noise ratio^21^, but we believe the simplicity of recording and replaying sound for haptic feedback can outweigh the aforementioned drawbacks. The perceived realism of audio-driven vibrations can be further enhanced by combining them with complementary visual and audio stimuli^22,23^, compensating for signal components outside the tactile–auditory overlap^4^. However, realistic vibrotactile feedback requires an in-depth understanding of the impact of stimulus location, stimulation parameters, and skin mechanics.

Fingerpad skin is highly sensitive to amplitude and frequency modulation^24,25^: its densely distributed mechanoreceptors enable precise detection of vibrotactile stimuli^26^. Many previous studies have shown that the spatial and temporal characteristics of vibrations significantly affect tactile perception on the fingertip. Serhat et al.^27^ showed that applying the same oscillating forces at different fingertip locations significantly alters the resulting dynamic response of the entire fingerpad. However, few studies have explored how changing the relative phase between vibrating elements affects how tactile feedback is perceived. Sakurai et al.^28^ found that the distance between two pins vibrating in opposite directions influences both sensitivity and perception. Later, Kuruki et al.^29^ discovered that low-frequency phase-shifted vibrations were perceived differently than in-phase counterparts. These studies did not include frequencies above 75 Hz.

**Fig. 1:**
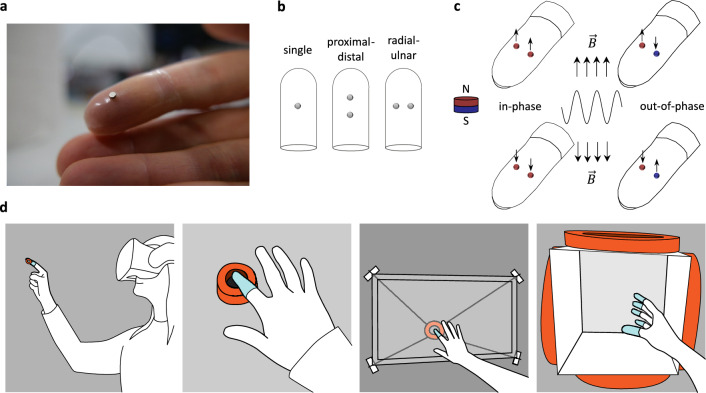
Different designs and potential actuation approaches for a wearable vibrotactile device comprising magnets embedded in a soft silicone sheath. (**a**) A single-magnet sheath worn on a fingertip^6^. (**b**) Three possible magnet placements: a single central magnet, two magnets arranged in the proximal-distal direction, and two magnets arranged in the radial-ulnar direction. (**c**) Radial-ulnar configuration with the magnets placed with the same polarity (in-phase vibrations) and opposite polarity (out-of-phase vibrations). When an oscillating magnetic field $\vec {B}$ is applied, each magnet experiences a force with or against the field, depending on its pole orientation. The north pole (N) of the magnet is shown in red, and the south pole (S) in blue. (**d**) Four ways in which magnets embedded in a soft finger sheath can be used to deliver vibrotactile feedback. For visibility, the sheaths are shown in pale blue and air coils in orange. From left to right: an air coil attached to or suspended above the fingerpad delivers haptic feedback of virtual object contact in virtual reality; a user wearing a sheath provides touchless two-dimensional motion input while feeling vibrotactile cues in response; a planar wire robot moves a coil behind a screen to track the user’s fingertip for touchless haptic feedback on a graphical user interface; a user wearing five sheaths reaches into a volume surrounded by large coils to feel and manipulate virtual objects.

Gertler et al.^6^ introduced a wearable fingertip device whose silicone rubber sheath contains a single miniature magnet (Fig. 1a) that is axially magnetized and actuated with a nearby air coil. This device achieved excellent mechanical coupling with the skin, generating clear localized vibration. However, natural touch typically engages a larger area of the fingerpad. To increase the effective surface area and try to create more realistic sensations, we redesigned the device by incorporating a second disk-shaped magnet. This modification introduces new degrees of freedom in the design, since the magnets can be placed in different locations (Fig. 1b) and oriented with either the same or opposite magnetic polarity (Fig. 1c). The present study examines two spatial arrangements of the magnets around the fingertip center: proximal-distal and radial-ulnar. Finite element analysis (FEA) of their dynamic response indicated that the placement of magnets in the radial-ulnar direction produced larger displacements than the proximal-distal direction for both polarity configurations. Consequently, fabrication and further investigation focused on this arrangement. A human-perception study was then conducted to evaluate the perceptual differences between vibrations produced by in-phase (same polarity) and out-of-phase (opposite polarity) sheath types (Fig. 1c). We hypothesized that the two types of vibrations would feel distinct at low frequencies but both be perceived as “in-phase” at high frequencies. Study participants also evaluated the device’s ability to render audio-driven haptic feedback of pre-recorded interactions with objects and surfaces. Finally, we describe a method to deploy this audio-based haptic rendering approach in virtual reality (VR). We envision several additional system designs (Fig. 1d) capable of generating a strong electromagnetic field at the user’s fingertip for touchless delivery of rich vibrotactile feedback.

**Fig. 2:**
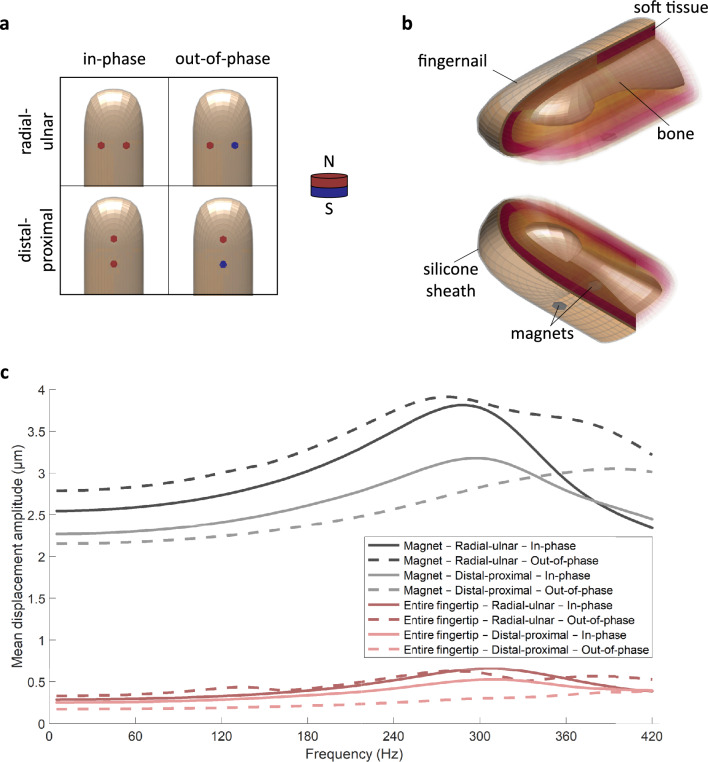
Tested FE models of the fingertip covered by a 215-$\mu$m-thick silicone sheath with two embedded magnets in (**a**) four configurations, given by the combination of magnet placement in terms of polarity (in-phase vs. out-of-phase) and position on the fingerpad (radial-ulnar vs. distal-proximal). The north pole (N) of the magnet is shown in red, and the south pole (S) in blue. (**b**) Isometric section views spotlighting the fingernail (top) and finger pad (bottom) regions of the FE model, including two magnets in the radial-ulnar configuration. (**c**) Average displacement FRFs of the magnets and the entire fingertip for different magnet arrangements and excitation frequencies in the range of 5–420 Hz.

## Results

### Simulation

The dynamic behavior of the fingertip covered by the silicone sheath with two embedded magnets is investigated via high-fidelity FEA; the simulation is based on DigiTip^30^ and was previously validated experimentally for a single-magnet sheath^6^. Figure 2a shows the four magnet configurations that were considered, corresponding to in-phase and out-of-phase polarities with radial-ulnar and distal-proximal placements. The magnets have diameters of 2 mm, and their centers are separated by 6.5 mm. The longitudinal distance between the magnets’ midpoint and the end of the fingertip is approximately 14.5 mm. Each model consists of 10,521 nodes and 9,088 linear hexahedral elements.

The details of the finite element (FE) model for the radial-ulnar magnet arrangement are depicted in Fig. 2b. The lateral half of the tissue layers (stratum corneum, epidermis, dermis, hypodermis, nail bed, and fingernail) are made transparent to reveal the complex internal structure and characteristic shape of the distal phalanx bone, which was assumed to be rigid due to its significantly higher elastic modulus. These anatomical features are modeled using the average dimensions reported for an adult female’s fingertip, while the external dimensions of the model correspond to the first author’s fingertip^6^. A 215-$\mu$m-thick external silicone layer encasing two magnets was added to the model as shown in Fig. 2b. The material properties and dimensions of the model components are reported in Tables S1 and S2, respectively.

For each of the explored magnet configurations, the frequency response functions (FRFs) are computed for displacements averaged over the magnets and also over the entire fingertip. The amplitude of the total force on each magnet is set to 1.471 mN based on experimental measurements. The analyses are performed using a frequency interval of 5 Hz across the range of 5–420 Hz. The FRF curves (Fig. 2c) demonstrate that magnet placement along the radial-ulnar direction generally results in higher overall displacement levels, which we speculate would cause higher perceived intensity. This configuration also leads to the largest magnet displacements across the entire frequency range when out-of-phase forces are applied. Therefore, we selected in-phase and out-of-phase magnets in the radial-ulnar configuration for further analysis.

To elucidate the properties that govern the forced dynamic responses of this fingertip system, we computed its natural frequencies and their corresponding mode shapes through free vibration analyses. Thirteen modes were detected in the considered frequency range of 0–420 Hz (Fig. S1), where the fourteenth one occurs at 436.4 Hz. The shape and order of the first seven modes match well with those previously calculated for the bare fingertip of an adult male^30^. The discrepancies in natural frequencies likely stem from dimensional differences and the presence of the sheath covering the fingertip.

The free vibration modes clarify the trends in the computed FRF curves (Fig. 2c). The first mode (143.9 Hz) involves global rotation around the phalanx bone and can be effectively excited by a moment obtained using out-of-phase magnets placed in the radial-ulnar direction. Thus, this magnet arrangement results in high mean fingertip deformation magnitudes around 140 Hz. The fifth mode (303.0 Hz) exhibits bulk in-out motion around the center of the finger pad, and it explains the resonance observed for in-phase magnet configurations around 300 Hz. The fourth mode (280.7 Hz) and ninth mode (383.0 Hz) involve opposite normal movement at the two lateral halves of the fingerpad. Since these modes can be effectively excited by out-of-phase forces applied to the magnets with radial-ulnar placement, they induce the peaks around 280 and 360 Hz in the mean fingertip displacement FRFs.

**Fig. 3:**
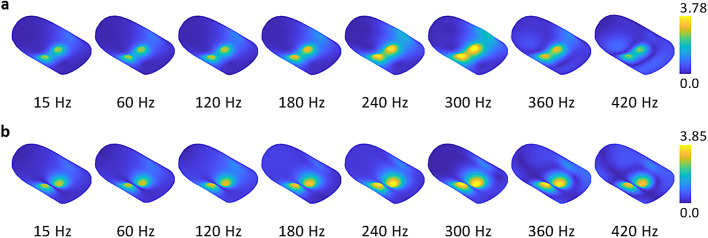
Displacement (in $\mu$m) contours of the fingertip excited at different frequencies. Harmonic normal forces are applied to two magnets with (**a**) identical or (**b**) opposite polarities (in-phase and out-of-phase configurations, respectively). A visual magnification factor of 500 is applied to the deformations to demonstrate the patterns clearly.

Finally, the displacement magnitude contours were computed to illustrate how the fingertip deforms under harmonic normal forces at particular frequencies. Figure 3 shows the results for (a) in-phase and (b) out-of-phase excitation at 15, 60, 120, 180, 240, 300, 360, and 420 Hz. For each excitation type, the contours are plotted on the same scale to assess the relative vibration levels. For both magnet configurations, the highest magnet displacement levels are observed at 300 Hz, confirming the computed FRFs (Fig. 2c).

**Fig. 4:**
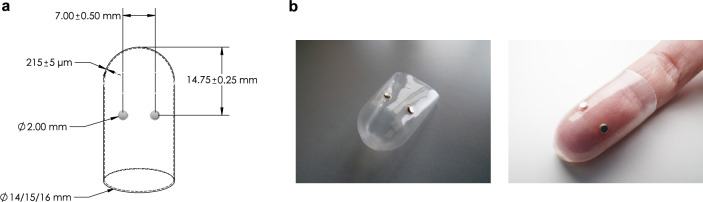
A soft silicone sheath with two embedded magnets in the chosen radial-ulnar configuration. (**a**) The dimensions of the overmolded sheath. (**b**) A fabricated sheath prototype removed from the mandrel (left) and worn on a user’s fingertip (right).

**Fig. 5:**
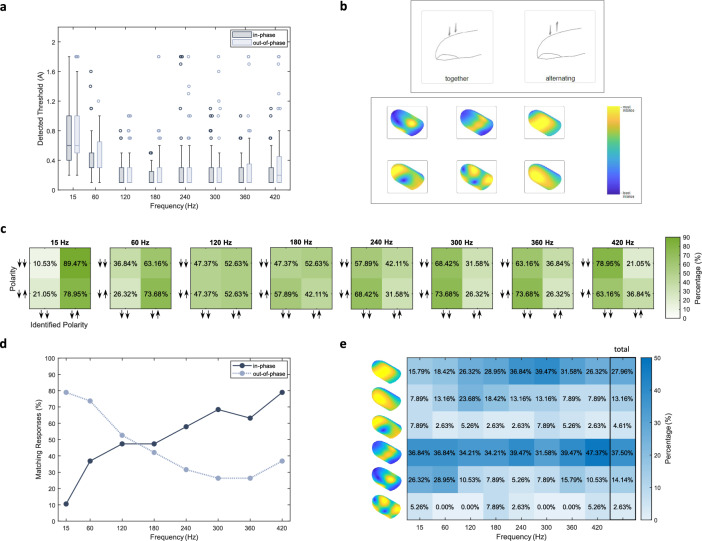
Results of the detection threshold and polarity and localization identification experiments. (**a**) Median, quartile, and outliers of the lowest input amplitude detected by all 24 participants across the eight tested frequencies for the in-phase (dark gray-blue) and out-of-phase (light gray-blue) configurations. (**b**) The choices given to participants for the polarity and localization identification experiment to define the type (top row; “together” or “alternating”) and the location (bottom row; six images with different distributions and a colorbar) of the perceived sensation on their fingertip. (**c**) Confusion matrices for the 19 participants representing the percentage of the identified polarity (x-axis: “together”, displayed as two arrows pointing in the same direction; and “alternating”, displayed as two arrows pointing in opposite directions) vs. the sheath configuration (y-axis: in-phase, displayed as two arrows pointing in the same direction; and out-of-phase, displayed as two arrows pointing in opposite directions) reported separately for the eight tested frequencies. (**d**) Percentage of responses correctly matched to the actual vibration type (e.g., choosing “together” while wearing an in-phase sheath is considered a match) across frequencies for in-phase (solid dark gray-blue line) and out-of-phase (dashed light gray-blue line) configurations. (**e**) A heat map showing the identified locations of the perceived sensation on the fingertip across different frequencies for both sheaths combined; the total column displays the percentages averaged over all frequencies.

### Soft magnetic fingertip device

Based on the simulation results, silicone rubber sheaths containing two miniature neodymium magnets placed in the radial-ulnar locations were fabricated in three different sizes (Fig. 4a) to accommodate variations in fingertip dimensions. For each size, two sheath types were produced: in-phase and out-of-phase, corresponding to magnets placed with identical or opposite polarities, respectively. The distance between the magnets and their location relative to the closed end of the sheath were consistent with the geometry of the FE model, design considerations, and the technical constraints related to the coil and finger dimensions, as described in the Methods section. The customized overmolding process used here produces a robust yet minimalist structure (Fig. 4b) capable of withstanding the oscillating magnetic forces applied to the embedded permanent magnets. These small haptic actuators, mechanically coupled with the user’s fingertip skin via the soft rubber, push and pull the surrounding tissue following the magnetic field lines.

### Human-perception study

We conducted a study with 24 participants to evaluate the perception of vibrations produced by in-phase and out-of-phase sheaths and to assess the quality of the tactile feedback provided by our device using sound recordings as the driving command. The study consisted of three experiments: detection threshold, polarity and localization identification, and interaction evaluation. The participants repeated all experiments twice and evaluated both magnet arrangements; the presentation order of the sheaths was balanced across participants. For all experiments described in this article, the user placed their fingertip beneath a coil to simplify the experimental setup, and they were never allowed to see the sheaths.

#### Experiment 1: detection threshold

This experiment evaluated the perception of in-phase and out-of-phase fingertip vibrations across a wide range of frequencies, and it also determined whether the type of vibration affects the detection threshold. The stimuli were 300-ms-long ramped sinusoids (starting and ending with 100 ms linear ramps) at eight frequencies (15, 60, 120, 180, 240, 300, 360, 420 Hz) designed to span human vibrotactile sensitivity and the key vibration modes identified through simulation. The command amplitude ranged from 0.05 V to 0.90 V (see the Methods section for more details). The stimuli at 15 Hz were not detected by one participant for either sheath type or by another participant wearing the out-of-phase version. Additionally, four participants did not detect thirteen stimuli at six frequencies, likely due to fatigue or the amplitude limits, as previously reported by Samuelle et al.^31^.

Figure 5a shows the distributions of the lowest detected input amplitude for each combination of participant and frequencies for both sheath types. Results indicated that frequency had a significant effect on detection threshold (F(7, 1098) = 93.41, p < 0.0001), and this influence notably depends on the order in which the sheaths were presented (frequency $\times$ order interaction: F(7, 1098) = 3.35, p = 0.0015). However, neither the sheath type (F(1, 1098) = 3.10, p = 0.0784) nor the presentation order (F(1, 22) = 1.11, p = 0.3026) alone had a significant main effect on detection threshold. When averaged over the levels of type and order, post-hoc pairwise comparisons showed that the detection thresholds at 15 Hz were significantly higher than the ones at 60 Hz (p < 0.0001), and these two frequencies resulted in significantly higher thresholds (p < 0.0001) than the other frequencies. Additionally, the threshold at 420 Hz was significantly higher than the one at 180 Hz (p = 0.0187).

Since the frequency $\times$ presentation order interaction was significant, we also compared frequencies for each order (averaged over sheath type, Fig. S2a). For the group who wore the in-phase sheath first (Fig. S2b), significant contrasts indicated that the thresholds at 240 Hz were lower than those at 60, 120, and 360 Hz (p = 0.0123, p = 0.0021, and p = 0.0062, respectively). For the group who wore the out-of-phase sheath first (Fig. S2c), 60, 120, 180, 240, 300, and 360 Hz led to significantly lower thresholds than 15 Hz (p = 0.0289, p < 0.0001, p < 0.0001, p = 0.0143, p = 0.0035, and p = 0.0012, respectively).

#### Experiment 2: polarity and localization identification

The objective of this experiment was to determine how participants perceive in-phase and out-of-phase vibrations across excitation frequencies. Specifically, the experiment sought to determine whether the magnets were felt as vibrating “together” or “alternating” and how the sensation was distributed over the fingerpad in terms of location and intensity (Fig. 5b). Figure 5c shows the confusion matrices representing the distribution of polarities identified by 19 participants for each stimulus separately. Figure 5d summarizes the percentage of responses that correctly matched the sheath type across the eight tested frequencies.

To test whether sheath type and excitation frequency affected the probability of a matching polarity response, we fitted a logistic generalized linear mixed model with participant as a random intercept. Frequency strongly influenced the response: relative to the reference frequency (15 Hz), higher frequencies were associated with substantially lower odds of an “alternating” response (120 Hz: OR = 0.10, p = 0.0131; 180 Hz: OR= 0.10, p = 0.0131; 240 Hz: OR = 0.06, p = 0.0027; 300 Hz: OR = 0.04, p = 0.0005; 360 Hz: OR = 0.05, p = 0.0012; 420 Hz: OR = 0.02, p < 0.0001). Neither the sheath type (OR = 0.41, p = 0.3591) nor the presentation order (OR = 0.88, p = 0.7947) significantly affected the responses. The random intercept variance was 0.78 (SD = 0.88), corresponding to an intra-class correlation of about 0.19, indicating approximately 19% of the total variance in the responses was due to differences between participants. These results show that, for both sheath types, the stimuli were perceived as “alternating” for the frequency range of 15–60 Hz and “together” for the range of 240–420 Hz.

Localization of the perceived sensation along the fingerpad across different frequencies is shown in Fig. 5e. The most prevalent choice across all frequencies was the image showing a narrow and continuous large-displacement region in the radial–ulnar direction (the fourth from the top). The image depicting two distinct high-amplitude areas in the radial–ulnar direction (the fifth from the top) was chosen more frequently at 15 Hz and 60 Hz. By contrast, the first image showing a wider skin movement region was chosen more often at higher frequencies, particularly around 180–360 Hz. These patterns were consistent for both sheath types (Fig. S3). Further statistical analysis was not possible due to the limited amount of data.

**Fig. 6:**
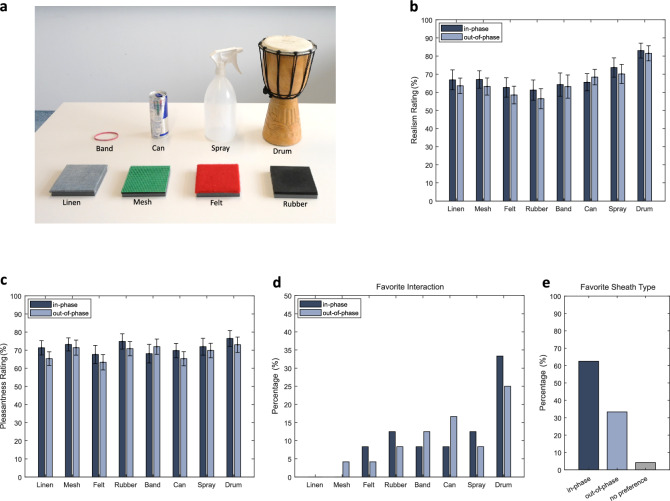
Results of the interaction rating experiment and final questionnaire. (**a**) Objects and surfaces presented to and rated by the 24 participants. Ratings (mean and standard errors) of (**b**) realism and (**c**) pleasantness of the vibrotactile feedback per video and sheath type (in-phase and out-of-phase shown by dark and light gray-blue, respectively). (**d**) The favorite interaction chosen by the participants for each sheath type. (**e**) The favorite sheath type chosen by the participants in the final questionnaire.

**Fig. 7:**
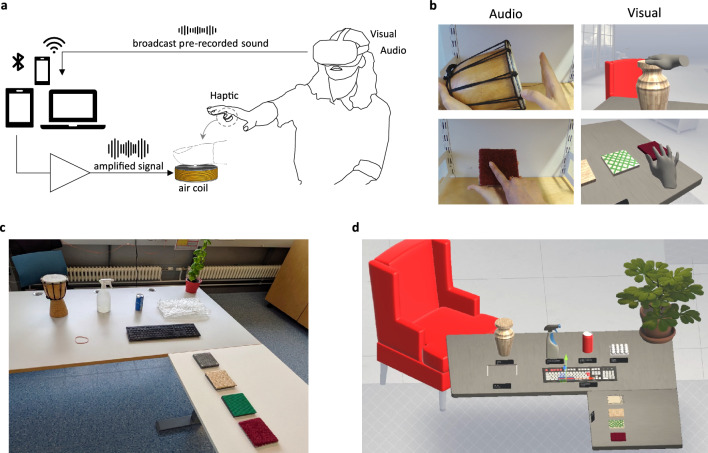
An example of how the sounds of physical interactions could be used to create haptic feedback in VR using our system. (**a**) High-level schematic showing how the sound output is converted into vibrotactile feedback: While the user wears the sheath and interacts with virtual objects, a pre-recorded or synthesized sound is broadcast from a VR headset to a media device such as a computer, tablet, or smartphone, then routed into an air coil via an amplifier. The amplified signal generates a magnetic field that acts on the embedded magnets and thereby produces the haptic feedback. (**b**) Virtual representations of two interactions similar to those used in the user study (tapping a djembe drum and sliding over a carpet surface). (**c**) Various objects used to record interaction sounds, and (**d**) a virtual scene that includes their digital twins.

**Fig. 8:**
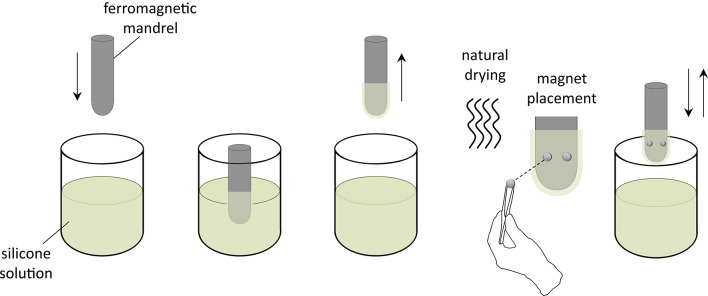
The overmolding process for fabricating a soft sheath with two embedded magnets. A ferromagnetic mandrel is immersed in a silicone solution to form the internal layers. After drying overnight at room temperature, two magnets are manually attached with the same or opposite polarity to the mandrel utilizing the magnetic attraction force. A thin layer of a silicone adhesive was applied on the surface of the magnets before attachment for additional support. Then, the mandrel is immersed again in the liquid silicone to create the external layers, embedding the magnets within the rubber.

**Fig. 9:**
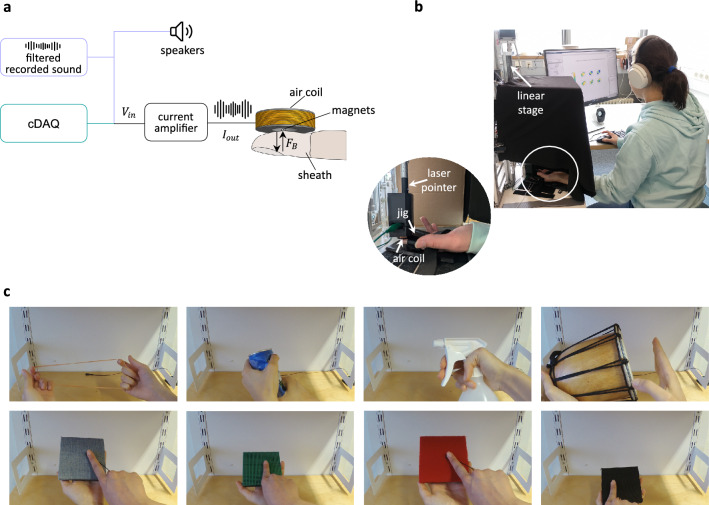
The experimental setup implemented for the user study. (**a**) Schematic of the hardware used for the three reported experiments, which all included haptic feedback; audio feedback from the speakers was provided only for the interaction rating experiment (lavender lines). The input signal for this experiment was generated by playback of an audio signal, while in the other two experiments it was produced by a data acquisition device (teal line). (**b**) A user wearing noise-canceling headphones while placing their finger in a custom jig that holds it stationary below an air coil hidden behind a black curtain. A laser pointer aligned with the coil’s central axis facilitated the correct placement of the fingertip under the coil. The coil was mounted to a linear stage to set the distance between its surface and the fingerpad. (**c**) Screenshots of the eight interactions used in the interaction rating experiment.

#### Experiment 3: interaction rating

This qualitative experiment aimed to evaluate the multimodal haptic experience provided by both versions of our device by asking participants to rate the audio-driven tactile feedback synchronized with eight pre-recorded videos showing interactions with four textured surfaces (labeled ‘Linen’, ‘Mesh’, ‘Felt’, and ‘Rubber’) and four objects (labeled ‘Band’, ‘Can’, ‘Spray’, and ‘Drum’) (Fig. 6a). The audio of the videos was transmitted to the device and speakers simultaneously via an audio divider. Participants rated the perceptual match, realism, and pleasantness of each interaction, as shown in Fig. 6b,c and Fig. S4 for both sheath types.

The drum-tapping video was rated as more realistic than most others (Drum - Linen: t(330) = 4.81, p = 0.0001; Drum - Mesh: t(330) = 4.84, p = 0.0001; Drum - Rubber: t(330) = 6.18, p < 0.0001; Drum - Band: t(330) = 4.85, p = 0.0001; Drum - Can: t(330) = 4.47, p = 0.0003; Drum - Felt: t(330) = 5.86, p < 0.0001). Additionally, the interaction with the spray bottle was rated as significantly more realistic than the one with the felt surface (Spray - Felt: t(330) = 3.17, p = 0.0356). These contrasts are averaged over type and order. The drum-tapping video was also rated significantly better than many videos for matching sensation (Drum - Linen: t(330) = 4.36, p = 0.0005; Drum - Mesh: t(330) = 5.34 p < 0.0001; Drum - Rubber: t(330) = 5.34, p < 0.0001; Drum - Band: t(330) =  5.02, p < 0.0001; Drum - Can: t(330) = 4.84, p = 0.0001; Drum - Felt: t(330) = 5.20, p < 0.0001).

The Aligned Rank Transform (ART) ANOVA indicated a significant video $\times$ order interaction for both realism and content-matching ratings. However, post-hoc pairwise comparisons between videos within each presentation order, after Tukey’s correction, did not yield any significant differences. Nevertheless, these results suggest that the overall preference for the drum video represents a consistent trend across the two devices and two presentation orders. After this rating task, the participants indicated their favorite video overall. Consistently, the interaction with the drum was the most frequently selected favorite for both sheath configurations, with a higher number of votes for the in-phase type (8/24 = 33.3%) compared to the out-of-phase one (6/24 = 25.0%) (Fig. 6d).

Although the video content had an overall effect on pleasantness (F(7, 330) = 2.21, p = 0.0332), post-hoc pairwise comparisons revealed no statistically significant differences for any pairs of videos after the adjustment for multiple comparisons (Tukey’s method). The sheath type exhibited a significant main effect on the pleasantness of the feeling (F(1, 330) = 5.56, p = 0.0190), suggesting that the rating differs across this parameter: the in-phase type had higher pleasantness ratings than the out-of-phase type (t(330) = 2.357, p = 0.019), averaged across video and presentation order.

#### Final questionnaire

After completing all tasks, the participants filled out a final questionnaire to indicate which sheath type they preferred. The in-phase sheath (15/24 = 62.5%) was favored over the out-of-phase one (8/24 = 33.3%), regardless of the presentation order (Fig. 6e); only one participant (4.2%) had no preference between the device configurations. Fourteen participants commented that they preferred the in-phase sheath because they felt stronger and/or clearer vibrations. Conversely, three participants who preferred the out-of-phase type mentioned that they felt the vibrations better, and four explained that the sensation was more enjoyable or better distributed. One participant commented that they grouped the vibrations as “buzzy” and “wobbly”, which correspond to being “together” and “alternating”, respectively. The most common themes in the responses to open-ended questions about the device and the experiment referred to the comfort of the sheaths, sweating, and fatigue or discomfort from the hand position (reported by 3, 4, and 6 participants, respectively).

### Implementation in VR

Here, we show how the presented device can be used to provide haptic feedback in VR. Figure 7a schematically describes the proposed technique. A user wears the device with an integrated air coil and interacts with virtual objects. The headset provides visual and audio feedback, while the sound is transmitted via a standard audio cable (either directly or by broadcasting it first to another media device such as a smartphone) to an amplifier, which is connected to the air coil. Consequently, the magnets push and pull the user’s fingertip skin in synchrony with the audio waveform. The main advantage of this approach is its simplified integration. The equipment and technical knowledge required to create the necessary haptic feedback are accessible and suitable for all levels of expertise; no new haptic content needs to be generated. Furthermore, this method can be employed to create plug-and-play hardware for other electromagnetic vibrotactile devices: short sound recordings can be used as the input command of such systems to render event-based haptic interactions with virtual representations of real objects.

Figure 7b-d and supplementary video SV1 demonstrate the integration of recorded audio that serves as the actuation command for our device in a VR application, thereby generating real-time haptic feedback. Sound recordings of ten interactions (Fig. S5) are replayed while the user wearing a VR headset with integrated hand tracking (Oculus Quest 1) explores, pokes, and squeezes virtual objects.

## Discussion

The design of wearable fingertip devices is important for delivering high-quality haptic feedback. This article presents advanced versions of a soft fingertip sheath^6^ with two rigid magnets placed along the radial-ulnar direction in two configurations, in-phase and out-of-phase. This device allows us to investigate the impact of magnet location and polarity on human perception in wearable vibrotactile devices. Additionally, the experimental platform was used to evaluate pragmatic audio-driven haptic feedback, using sound recordings as a minimalist input command to create realistic vibrotactile experiences.

The FRF results (Fig. 2c) showed fingertip and magnet displacement peaks between 270 and 310 Hz for both polarity arrangements with radial-ulnar magnet placement. These results conform with the well-known vibrotactile detection-threshold curve, which shows a maximum sensitivity around 250 Hz^32^, and they also align with those from the detection threshold experiment, where the highest sensitivity was found in the range of 120–360 Hz (Fig. 5a). This experiment was designed to establish detectability rather than to measure thresholds precisely. Hence, we began testing at relatively high amplitudes and used coarse amplitude steps, which reduced resolution in the mid-frequency band; starting at lower amplitudes with finer increments would yield more precise threshold estimates but would substantially increase testing time and participant burden. However, both sheath types successfully produced easily detectable vibrations even near the lower boundary of the human tactile frequency range, with a tendency toward lower thresholds for the in-phase configuration. Even though two participants could not detect the 15 Hz sinusoid at any tested magnitude, they did perceive this frequency in the polarity and localization experiment. Both the simulation and the detection threshold results are supported by the localization identification experiments, in which participants tended to choose an image showing a larger, more intensely affected area on the fingerpad for 240–360 Hz (Fig. 5e).

Notably, excitation frequency dominated perception, outweighing the influence of magnet polarity; participants mostly defined the low-frequency vibrations (15–60 Hz) as “alternating” and the higher-frequency vibrations (240–420 Hz) as “together”. We hypothesize that phase shifts between high-frequency vibrations are less perceivable than phase shifts between low-frequency vibrations, causing high-frequency stimuli to be perceived as “together”. Furthermore, the perception of both magnet polarities as “alternating” at low frequencies did not match our hypothesis of correct polarity perception in this range. Two design constraints of the experimental setup may have increased task difficulty and impaired participants’ ability to distinguish the actuators’ vibration patterns.

First, the position of the magnets relative to the coil’s effective magnetic range should be considered. Specifically, the magnetic field of a core-less electromagnet becomes less uniform with increasing distance from the center of the coil. Thus, magnets that are not precisely aligned with the coil’s central axis undergo lateral forces and torques in addition to the expected axial force. Second, the rotational effect is further amplified due to the placement of the magnets on the curved fingertip surface, which would not be perfectly perpendicular to the magnetic field even if a uniform magnetic field was achieved. These phenomena likely generate skin vibrations more complex than those considered in the simulation. A possible solution could be to replace the coil with two smaller ones (one per magnet), creating more localized and controlled magnetic fields at the cost of hardware complexity. Beneficially, different waveforms could then also be played in each location.

While acceleration measurements are commonly used as the input for vibrotactile feedback, audio recordings could be a good alternative when their frequency content overlaps with the haptic sensitivity range^22^. However, frequency overlap is not the only factor to consider; effective haptic feedback also requires tactile-auditory congruence and context. For instance, in string instruments such as an acoustic guitar, contrabass, or cello, the finger pressing the string vibrates at the same frequency as the produced sound. In this case, the recorded audio both lies within the haptic range and closely reflects the acceleration applied to the finger. In contrast, for keyboard instruments, such as a piano, the tactile and auditory experiences do not align. While pressing a key, the finger primarily senses the mechanical forces arising from dynamic contact, while the sound emitted by resonating strings is physically decoupled from the finger. Consequently, keys feel largely similar regardless of the acoustic note produced, and higher-octave notes (>1,000 Hz) exceed the haptic sensing range. In such cases, audio-driven vibrotactile feedback may result in mismatched or unrealistic sensation. To avoid effects from audible-only frequencies, we applied a 1,000 Hz low-pass filter to the recorded audio, so that only sound components within the auditory-tactile range remained in the input signal. Mild environmental noise within the vibrotactile range is difficult to avoid but may still allow audio recordings to yield convincing haptic feedback^4^.

Our results indeed suggest that contextual plausibility can override imperfect tactile–auditory correspondence. The interaction with the djembe drum was rated as both the favorite and the most realistic across the two configurations and both presentation orders. This instrument produces sound primarily between 70 Hz and 800 Hz, a range that falls entirely within the tactile-auditory overlap (Fig. S6) with strong components denoted by the membrane vibrations between 400 Hz and 800 Hz^33^. However, the effect of the primary frequency range of different musical instruments on tactile perception should be further explored. The absence of significant pairwise differences between the drum and the other videos within each presentation order may reflect limited statistical power within the sheath type subgroups or variability across the hardware (e.g., differences in sheath tightness around the finger). Interestingly, the interaction with the spray bottle was ranked second, although its audio was dominated by the hissing sound of pressurized water, which is not directly related to the contact between the finger and the trigger. In future work, comparing vibrotactile feedback generated from accelerometer measurements and from audio recordings would enable researchers to assess perceptual equivalence and determine the situations where audio can serve as a viable driving input for haptic feedback.

Generally, participants rated the haptic feedback of object-based interactions as more realistic and better matching the video content than interactions with textured surfaces. A possible explanation is that most of the sound recorded for the textures was caused by contact with the rigid microphone, while the object-based interaction recordings captured the sound produced by the finger itself. By contrast, neither object-based interactions nor textured surfaces showed an advantage in perceived pleasantness. In summary, the current implementation of our system appears more suitable for event-based interactions^34^ rather than for texture rendering, which would require a more sophisticated modulation of the input signal based on user movement^23,35^. For momentary interactions, however, the system reliably produces credible vibrotactile feedback that can be integrated into VR environments. In such applications, the coil position should be fixed relative to the fingertip, either by attaching the air coil to the sheath as shown in Fig. 1d (e.g., using a silicone adhesive^6^) or by attaching a stronger iron-core coil to the fingernail.

Overall, most participants favored the in-phase sheath; although quantitative comparisons did not show a statistically significant difference between the configurations, participants consistently reported feeling stronger vibrotactile feedback when the two magnets had the same polarity. Interestingly, this comment does not match the mean magnet and fingertip displacements predicted by our simulation at most frequencies, nor does it correspond with the measured detection thresholds, which showed no significant differences between sheath types, highlighting the complexity of vibrotactile feedback. Considering the results of the polarity and localization experiment, and given the additional complexity of fabricating and handling out-of-phase sheaths due to the attraction force between the magnets, the in-phase type appears more advantageous. Systematically exploring different magnet placements across the fingertip and their effects on dynamic response and perception could facilitate optimizing the design of similar vibrotactile devices.

## Methods

### Fabrication of soft sheaths with embedded magnets

The sheath was fabricated in three sizes using a dip molding technique similar to that described previously by Gertler et al.^6^, who embedded a single magnet in a thin rubber sheath. Here, the addition of a second magnet posed new challenges, as the magnets tended to move towards each other while the fresh silicone layers were drying. Furthermore, the magnets can attach to one another when the cured sheath is removed from the mandrel or while the sheath is being donned or doffed, tearing the rubber. To address these issues, we increased the overall sheath thickness, reduced the dimensions and strength (grade) of both magnets, and secured each magnet in its position with a thin layer of adhesive. These key modifications kept the overmolding process practical, preserved the minimalist design, and ensured fabrication of functional sheaths.

The overmolding process (Fig. 8) involved immersing a cylindrical ferromagnetic mandrel (diameter of 14 mm, 15 mm, or 16 mm) with a hemispherical end into silicone (Dow Silicones Corporation, USA) four times to produce the internal layers, followed by overnight drying. Next, a silicone adhesive (Elastosil, Wacker Chemie AG, Germany) was applied to cylindrical permanent neodymium magnets (grade N45 with a diameter of 2 mm and height of 0.7 mm) on the surfaces facing the mandrel. On the coated mandrel, each magnet pair was manually placed in the radial-ulnar arrangement with a center-to-center distance of 7 ± 0.5 mm, approximately 15 mm from the curved end of the mandrel (Fig. 4a). The distance between the magnets was chosen to prevent magnetic interaction and overlapping vibrations^28^. After the silicone adhesive cured at room temperature for about 45 min, the mandrel was immersed four more times to form the external layers and then was placed in an oven set to 70°C for 8 hours to stay below the magnets’ demagnetization temperature during the curing process. Finally, the cured sheath was removed from the mandrel and cut to the desired length (30 mm). The overall thickness of the eight-layer sheath was 215 ± 5 $\mu$m.

### Sound and video recordings

Two types of interactions were recorded for use in the perceptual study (supplementary video SV2) and implementation in VR (supplementary video SV1): continuous sliding over a textured surface and momentary contact with an object (e.g., tapping a drum or squeezing a spray bottle). All interactions were filmed with a Zoom Q8 video camera (ZOOM Corporation, Japan). For videos depicting interactions with textured surfaces, audio was recorded using a microphone (Lavelier II, RøDE Microphones Ltd., Sydney, Australia) held beneath the finger, as the sound levels produced by finger contact alone were too low to be recorded effectively. Therefore, the recorded sound mainly captured the surface’s dynamic contact with the microphone rather than the finger itself. For videos showing object-based interaction, audio was captured using the camera’s integrated microphone. The objects and corresponding contact scenarios were selected to produce sounds that were partially or fully within the tactile frequency range, with varying levels of environmental noise (i.e., sounds that were not directly related to the interaction but occurred as side effects or were produced by an external source). In all videos, each interaction was repeated three times. The audio signals were filtered with a second-order Butterworth low-pass filter with a cutoff frequency of 1000 Hz to match the human vibrotactile sensitivity range. The filtered audio signals were verified to remain within the range of ± 1.

### Human-perception study

The study was approved by the Ethics Council of the Max Planck Society under the Haptic Intelligence Department’s framework agreement as protocol F036A. All experiments were performed in accordance with the relevant ethics guidelines of the Max Planck Society and in accordance with the Declaration of Helsinki. All participants provided informed consent, and those not employed by the Max Planck Society were paid 12 euros per hour (on average 18 euros for their participation time of 90 minutes).

#### Participant information

A total of 24 participants (14 female and 10 male, 20 right-handed and 4 left-handed) with ages ranging from 18 to 46 years (M = 31, SD = 7) took part in the study. The participants had at least an upper-intermediate level (CEFR-B2) of English proficiency and widely varying levels of familiarity with haptic devices. In the polarity and localization identification experiment, the stimuli were inadvertently not fully randomized for 5 participants, so these responses were excluded from the analysis to avoid order-effect bias. The final sample for that experiment thus included 19 participants (12 female, 7 male; 15 right-handed, 4 left-handed) who had the same age range of 18–46 years (M = 30, SD = 6).

#### Experimental setup

The study was conducted in a controlled laboratory setting. The actuation system comprised a DC power supply, a linear current amplifier (based on a high-power operational amplifier in a transconductance circuit with a gain of 2.0 A/V through a 0.5 $\Omega$ power resistor; Fig. S7), an air coil, and one of the developed sheaths. The choice of the coil dimensions and electromagnetic properties is critical because these parameters highly affect the resulting vibrations. We used a commercially available air coil (LU32/068/071, I.T. Intertechnik Kerpen GmbH, Germany) with a resistance of 719 m$\Omega$ and an inductance of 685 $\mu$H at 500 Hz. The external and internal diameters of the coil were 32 mm and 15 mm, respectively. The internal diameter was selected to encompass the majority of a typical fingerpad’s surface area and to keep the magnets within the magnetic field. To simplify the experimental setup, each participant’s fingertip was positioned beneath the coil. The coil was mounted on a linear stage to align the magnets with its surface. A laser pointer was placed directly above the center of the coil to facilitate correct positioning of the fingertip and the magnets.

The input signal was generated either by a data acquisition device (NI cDAQ-9171 and NI 9264, National Instruments Corp., USA) or by playback of a recorded audio signal through the computer’s on-board sound driver (Realtek(R) Audio), depending on the experiment (Fig. 9a). In the latter case, an audio divider enabled simultaneous presentation of the tactile feedback through the device and audio feedback through speakers. The actuation system was placed behind an opaque curtain to conceal the sheath and prevent visual cues related to the actuation (Fig. 9b). The setup also included a computer, monitor, numeric keypad, mouse, keyboard, a custom 3D-printed jig to secure each participant’s hand to the table in a stable position, and noise-canceling headphones to minimize auditory distractions in the first two experiments.

#### Procedure

Each participant took part in three experiments for each sheath type. They could take short breaks between experiments and were encouraged to take a longer break before putting on the second sheath. After filling out a standard demographic questionnaire, the participant was asked to wash and dry their hands. Then, the experimenter measured their dominant index finger to select a suitable size (see supplementary text for more information) and placed the sheath on their fingertip behind a black curtain, ensuring that the magnets were symmetrically located with respect to the center of the fingerpad. The following steps were repeated for each sheath type: the experimenter measured the distance between the magnet centers as well as the longitudinal distance between the point at the center of the magnets and the distal end of the fingertip to ensure that the selected sheath had been fabricated and donned correctly. The participant’s fingertip was then placed in the jig and aligned with the center of the air coil using the laser pointer (Fig. 9b). Once correct positioning was verified, the participant’s hand was secured with a strap of hook-and-loop fasteners to keep the correct alignment throughout the three experiments. The vertical position of the coil relative to the fingertip was adjusted using a linear stage: the coil was incrementally lowered until it contacted the finger and was then lifted one millimeter to align the magnet surface with the coil’s surface (Fig. 9b).

In the detection threshold experiment, we tested 300-ms-long sinusoids that included 100-ms-long ramps at both the beginning and end of the stimulus at eight frequencies. This duration was chosen to exceed the minimum required for detection^36^. The participants were asked to press the “enter” key on a numeric keypad as soon as they felt a vibration, as in widespread audiometry tests. The command amplitude for each frequency began at 0.10 V and was increased by 0.10 V increments until either it reached 0.90 V or the first detection occurred; the corresponding input current started from 0.2 A and ranged from 0.2 to 1.8 A with an increment of 0.2 A. Then, the precise detection threshold for the tested frequency was determined through a fine adjustment by decreasing the detected amplitude by 0.05 V once. The order of the frequencies followed a tactile-adjusted version of the guidelines for threshold audiometry^37^: 180, 240, 300, 360, 420, 180, 15, 60, and 120 Hz. The delay between successive vibration stimuli varied randomly between 1 and 4 seconds. The participants’ responses were constantly monitored to ensure synchronization with the actuation and identify any false positive reactions. The participants repeated the complete test three times, separated by brief breaks. The data from the first presentation of the 180 Hz sinusoid in each test were discarded.

The polarity and localization identification experiment tested sinusoids at the same frequencies used in the detection threshold experiment, with a relatively large amplitude of 1 V (2 A) and a duration of 1 s. As before, each sinusoid started and ended with a 100-ms-long linear amplitude ramp, and the presentation order was randomized. Participants initiated each stimulus by clicking a button on a graphical user interface. After the stimulus played, they were asked to determine whether the sensation felt as though the magnets were moving “together” or “alternating” (Fig. 5b). After this selection, the same sinusoid was replayed, and they were asked to select the image that best represented the distribution of the vibrotactile sensation on their fingertip (Fig. 5b). The six presented images resembled the low-order natural vibration modes found by the FEA (Fig. S1) and were used for illustration purposes only.

In the interaction rating experiment, the participants watched eight short (3–9 s) videos in random order while receiving audio-driven vibrotactile feedback on their fingertip. The participant played the video at their convenience and could replay it as many times as needed. After each video, they rated their experience on a Likert scale ranging from “strongly disagree” to “strongly agree” for three aspects: (i) match between the perceived sensation and the video content (“the feeling on my fingertip matched the video”); (ii) realistic representation of the interaction (“the feeling on my fingertip was realistic”); and (iii) pleasantness (“the feeling on my fingertip was pleasant”). Four out of eight videos showed a finger sliding horizontally along textured surfaces: linen, plastic mesh, felt, and wavy rubber. The other four videos depicted momentary interactions with different objects: pulling a rubber band, crumpling an empty soda can, squeezing a spray bottle filled with water, and tapping a djembe drum (Fig. 9c). Each interaction, whether with a texture or an object, was repeated three times in the video. After rating all eight videos, the participant was asked to select their favorite and explain their choice.

Finally, after completing the experiments with both sheath types, participants filled out a post-study questionnaire. They were asked to indicate their preferred sheath type (identified as the first or second sheath) and explain their choice. Additionally, they responded to open-ended questions regarding the sheath design, wearability, tested hardware and setup, and overall study experience.

#### Data analysis

All analyses were conducted in R (version 4.4.3). The significance level was set at $\alpha$ = 0.05 for statistical tests. For the detection threshold and interaction rating experiments, we used a type III non-parametric mixed-design Aligned Rank Transform (ART)^38^ ANOVA (N = 24). ART ANOVA was selected because our data were non-normally distributed and showed heteroscedasticity. Specifically, for the detection threshold experiment, the model tested the effects of frequency (8 levels), type (same polarity/in-phase vs. opposite polarity/out-of-phase), and presentation order of the device’s configurations on the detection threshold. Prior to the analysis, missing values were imputed by replacing them with the maximum tested amplitude $T_{\max }\,=0.9\,V$, following the approach of Cargano and Plack^31^. We chose this conservative approach since all participants were able to detect the stimuli at 1 V (2 A) in the polarity and localization experiment, and deleting all trials for missing data could lead to biased means and standard errors. Additionally, sensitivity analyses produced consistent results: key contrasts and significance patterns were unchanged between robust linear mixed-effects model runs with the original data and with $T_{\max }$ imputed data. For both experiments, post-hoc pairwise comparisons were conducted using the ART procedure with the Kenward-Roger method for approximating the degrees of freedom and with Tukey’s method to adjust the p-values.

For the polarity identification experiment, we fitted a generalized linear mixed model with a binomial logit link using glmer (lme4) to model the binary outcome (polarity). The model specification was:

1 $$\begin{aligned} Polarity \sim Type *Frequency + Order + (1 | ParticipantID). \end{aligned}$$

Participants were included as a random intercept (N = 19, n = 304). The model used the Laplace approximation for estimation. The Bobyqa optimizer was set via glmerControl to improve convergence. The fixed effects were evaluated using Wald z-tests. Post-hoc pairwise contrasts were computed with emmeans and Tukey’s adjustment. Model diagnostics indicated an adequate model fit, the Pearson dispersion ratio was close to one ($\approx$1.07), and DHARMa residual checks showed no evidence of misspecification (p = 0.205).

## Supplementary Information

Supplementary Information 1.

Supplementary Information 2.

Supplementary Information 3.

## Data Availability

The datasets used and/or analyzed during the current study are available from the corresponding authors on reasonable request.
