# Supplementary material for: Comparing placement and polarity configurations of a two-magnet fingertip vibrotactile device
Source: Sci Rep. 2026 Mar 8;16:12600. doi: 10.1038/s41598-026-41307-7 (PMC13086944; doi:10.1038/s41598-026-41307-7)
Supplement: Supplementary file 1 — Supplementary Information 1. [file 41598_2026_41307_MOESM1_ESM.pdf]

Supplementary Information for

Comparing Placement and Polarity Configurations of a Two-Magnet Fingertip Vibrotactile Device

Ifat Gertler[1,2], Giulia Ballardini[1], Demet Tangolar[1], Gokhan Serhat[1,3*], and Katherine J. Kuchenbecker[1,2*]

[1]Haptic Intelligence Department, Max Planck Institute for Intelligent Systems, Heisenbergstr. 3, 70569 Stuttgart, Germany
[2]Faculty of Engineering Design, Production Engineering and Automotive Engineering, University of Stuttgart, Pfaffenwaldring 9, 70569 Stuttgart, Germany
[3]Department of Mechanical Engineering, KU Leuven, Spoorwegstr. 12, 8200 Bruges, Belgium
[*]Corresponding author emails: gokhan.serhat@kuleuven.be, kjk@is.mpg.de

Other supplementary materials for this manuscript:
Supplementary video SV1
Supplementary video SV2

Supplementary Text

For each participant, the appropriate sheath size was determined according to the measured width of their finger: participants with a finger width below 14 mm (13.65 ± 0.57 mm) were given a 14-mm-diameter sheath, participants with a finger width between 14 mm and 15.5 mm (15.21 ± 0.46 mm) were given a 15-mm-diameter sheath, and participants with a finger width above 15.5 mm (16.28 ± 0.65 mm) were given a 16-mm-diameter sheath.

The placement of the magnets was measured for each sheath worn by a participant. The distance between magnet centers was 6.8 ± 0.3 mm for the in-phase configuration and 6.7 ± 0.2 mm for the out-of-phase configuration. The longitudinal distance from the magnet centers to the distal end of the fingertip was 14.6 ± 0.8 mm for both configurations.

Supplementary Tables

Table S1: The values of the elastic modulus, Poisson's ratio, and density for the silicone sheath, the magnet, and the six fingertip tissue layers, adapted from Serhat and Kuchenbecker [1] and Gertler et al. [2].

	**Elastic modulus (MPa)**	**Poisson's ratio**	**Density (g/cm^3)**
Sheath	1.39	0.49	1.12
Magnet	160 000	0.24	30.61*
Stratum corneum	1.000	0.30	1.100
Epidermis	0.140	0.40	1.106
Dermis	0.080	0.40	1.274
Hypodermis	0.034	0.48	0.920
Nail bed	1.000	0.30	1.100
Fingernail	170.0	0.30	1.300

* The density was calculated as the ratio of the actual magnet mass (0.0167 g) to the total volume of the magnet elements in the FE model (0.546 mm^3). In the hexagonal prism-shaped discretized magnet model, the length of the longest base diagonal corresponds to the actual magnet diameter (2 mm), while the height is taken to be equal to the sheath thickness (0.215 mm) to optimize mesh fidelity.

Table S2: The dimensions of the model shown in Fig. 2b. The values marked with an asterisk (*) were measured using calipers. The other values were sourced from the references and relationships provided by Serhat and Kuchenbecker [1] for an average female index finger, if applicable.

**Variable**	**Value (mm)**
Exterior sheath thickness	0.215*
Thickness of dermis	1.5
Fingertip length	25.0*
Soft tissue thickness at the interphalangeal joint	2.7
Fingertip width	15.1*
Bone length	20.1
Fingertip height	11.3*
Bone length at the distal section	8.3
Total nail length (apparent + root)	9.8* + 4.6 = 14.4
Bone width at the distal section	7.7
Nail width	9.4*
Bone height at the distal section	3.7
Nail height	2.5*
Bone width at the proximal section	9.5
Nail thickness	0.4
Bone height at the proximal section	6.0
Thickness of stratum corneum	0.4
Bone width at the neck	4.4
Thickness of epidermis	0.1
Bone height at the neck	3.2

Supplementary Figures

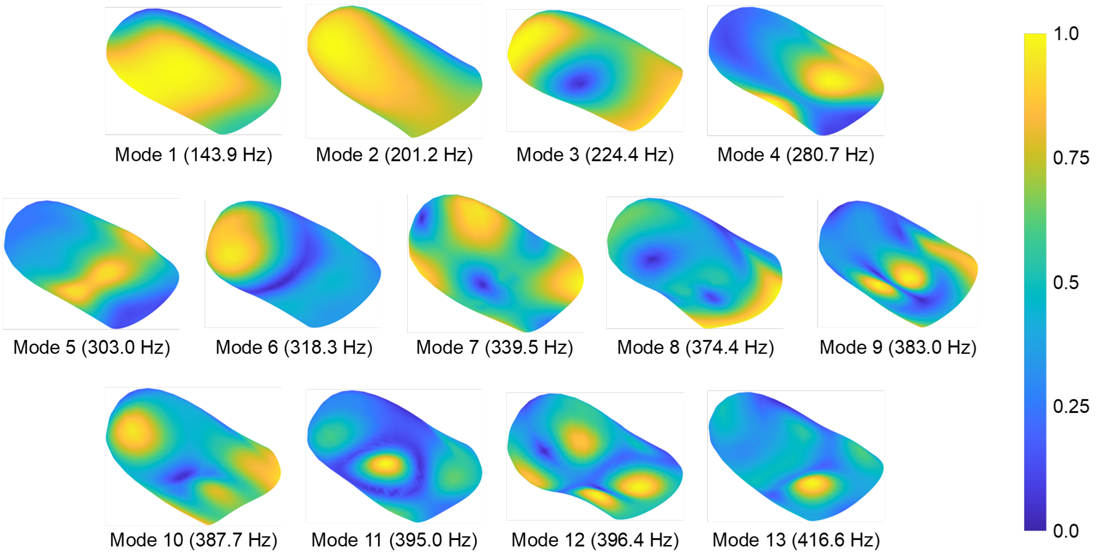

Figure S1: First thirteen free-vibration modes of the fingertip covered by the silicone sheath with two embedded magnets placed in the radial-ulnar configuration. The color bar values show normalized displacement.

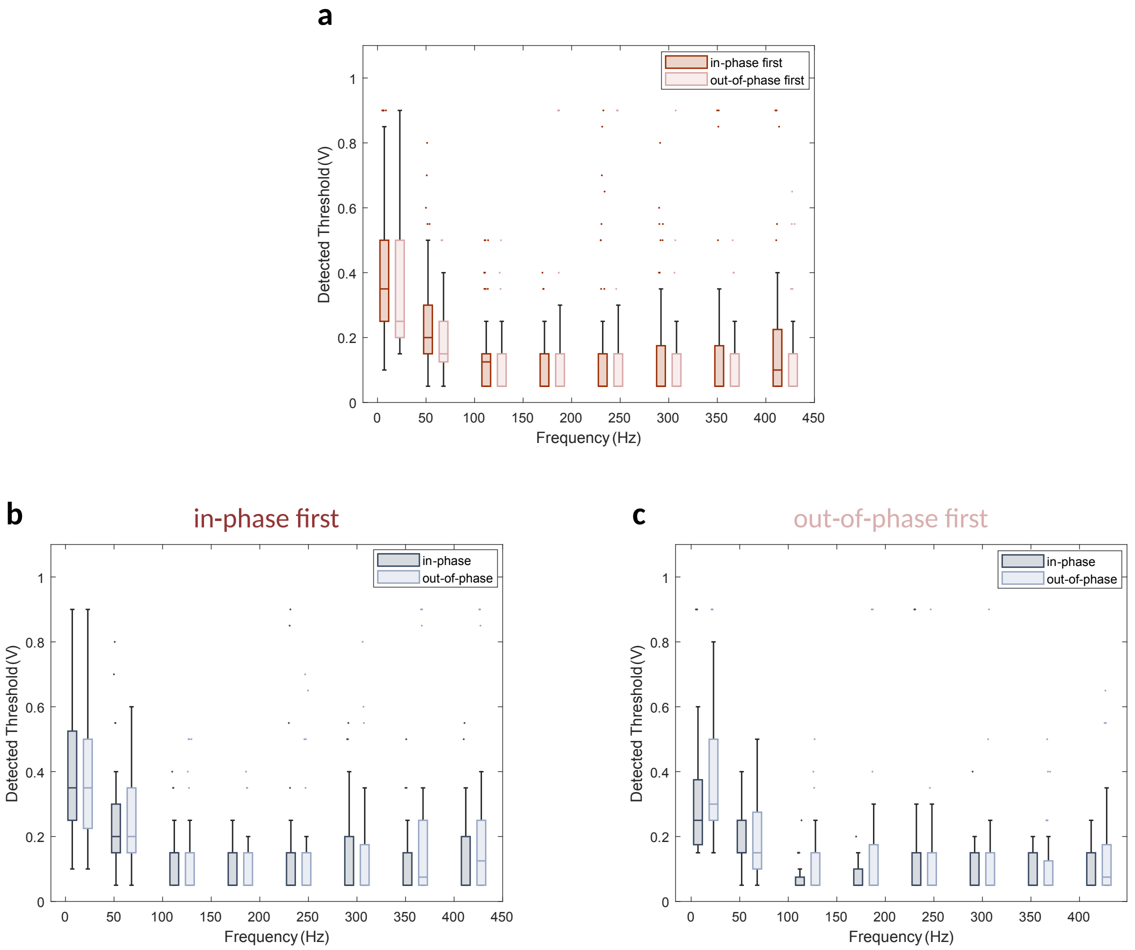

Figure S2: Additional human-perception study results for the detection threshold experiment. Detection thresholds across frequencies grouped by (a) presentation order (in-phase first and out-of-phase first), and by (b) in-phase first and (c) out-of-phase first presentation order.

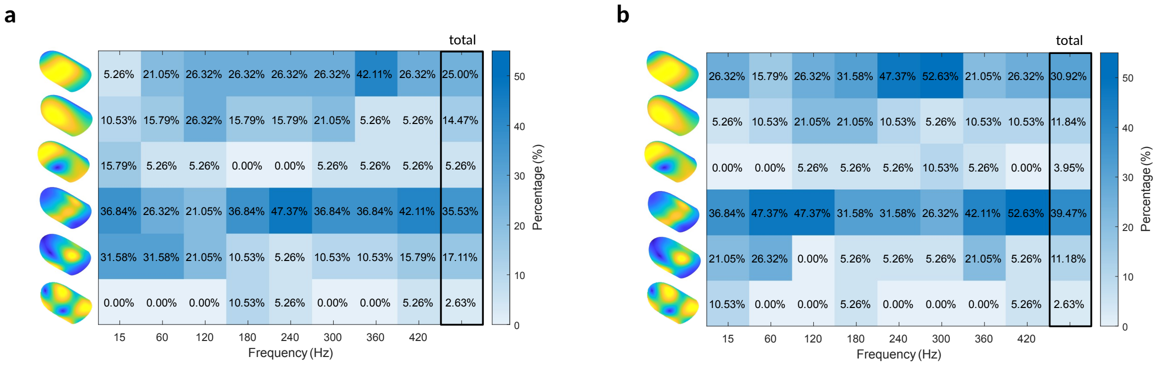

Figure S3: Additional human-perception study results for the polarity and localization identification experiment. Each participant chose the image that best corresponded to the sensations they felt on their fingertip at each frequency, with yellow indicating strong sensation. Heat maps showing the distribution of perceived sensations for participants wearing (a) in-phase and (b) out-of-phase sheaths across the tested frequencies. The cell color is proportional to the rating percentage.

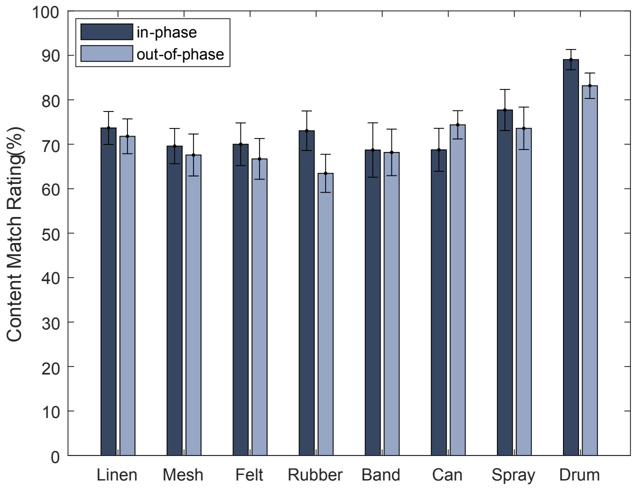

Figure S4: Additional human-perception study results of the interaction rating experiment. Mean and standard error of video rating results for the question of how well the feeling on their fingertip matched the video content.

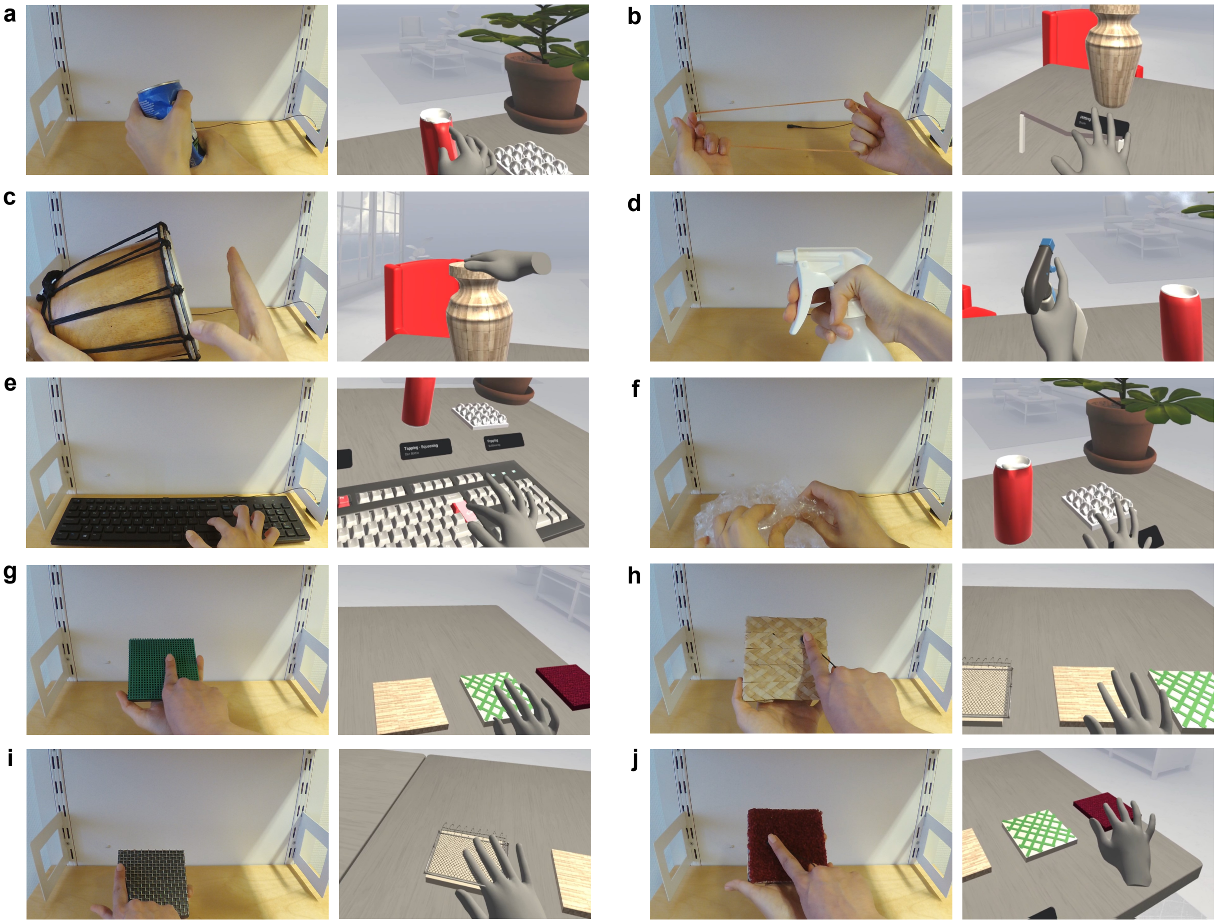

Figure S5: Real-life interactions with objects and surfaces and their virtual representation: (a) crumpling an empty drinking can, (b) pulling a rubber band, (c) tapping a drum, (d) squeezing a spray bottle, (e) hitting a keyboard, (f) popping a piece of bubble wrap, and sliding a finger over (g) a plastic mesh, (h) wicker, (i) a metal mesh, and (j) carpet.

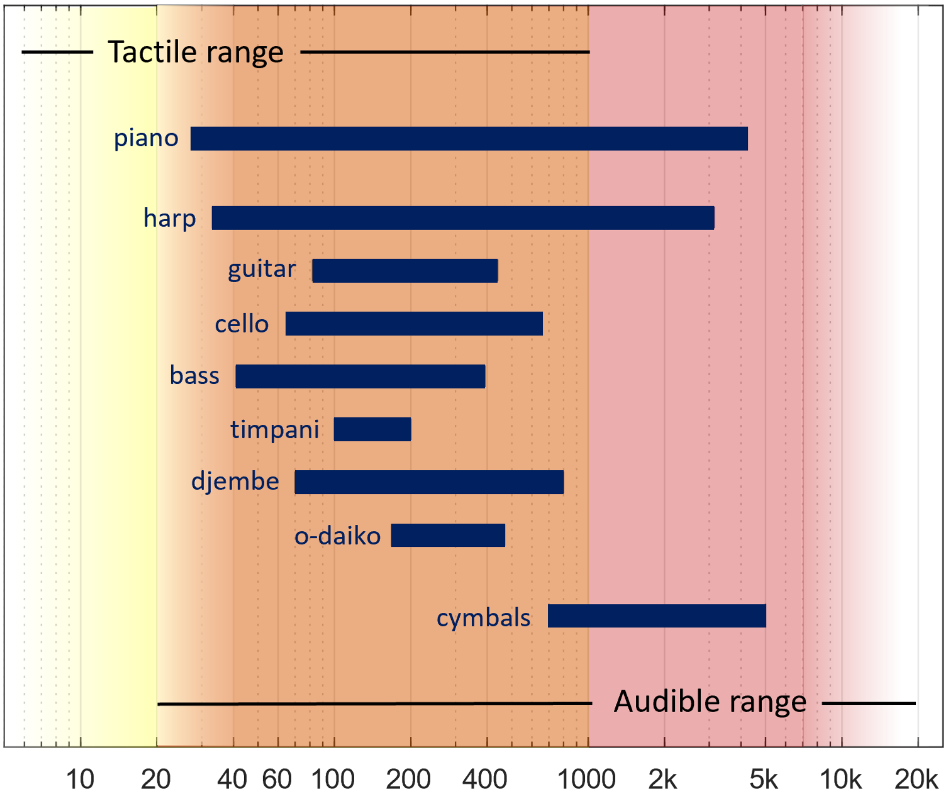

Figure S6: Tactile–auditory overlap and primary frequency ranges [3, 4, 5] of selected musical instruments.

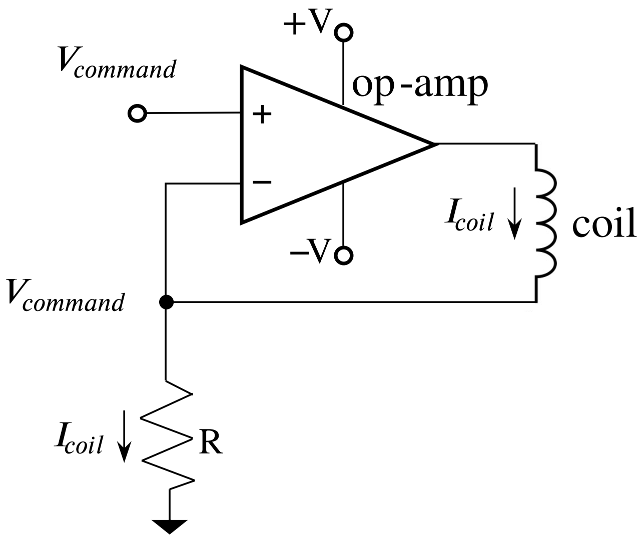

Figure S7: Linear current amplifier circuit ($R = 0.5\,\Omega, V = 15\,\text{V}$) used for the detection threshold and polarity and localization experiments. The high-power operational amplifier (op-amp) is an STMicroelectronics L165, and the resistor has a power rating of 4 W. Because the op-amp is configured in negative feedback, one can assume that the voltages at the non-inverting and inverting inputs are equal and that no current flows into or out of either input. Thus, the circuit causes a current $I_{coil} = V_{command}/R$ to flow through both the coil and the power resistor.

References

[1] G. Serhat and K. J. Kuchenbecker, "Free and forced vibration modes of the human fingertip," *Applied Sciences*, vol. 11, no. 12, p. 5709, 2021.

[2] I. Gertler, G. Serhat, and K. J. Kuchenbecker, "Generating clear vibrotactile cues with a magnet embedded in a soft finger sheath," *Soft Robotics*, vol. 10, no. 3, pp. 624–635, 2023.

[3] T. D. Rossing, "Acoustics of percussion instruments: Recent progress," *Acoustical Science and Technology*, vol. 22, no. 3, pp. 177–188, 2001.

[4] T. D. Rossing, *Springer Handbook of Acoustics.* Springer Science & Business Media, 2007.

[5] J. Meyer, "Tonal characteristics of musical instruments," in *Acoustics and the Performance of Music: Manual for Acousticians, Audio Engineers, Musicians, Architects and Musical Instrument Makers*, pp. 45–128, Springer, 2009.
